# Novel Antioxidant Properties of Doxycycline

**DOI:** 10.3390/ijms19124078

**Published:** 2018-12-17

**Authors:** Dahn L. Clemens, Michael J. Duryee, Cleofes Sarmiento, Andrew Chiou, Jacob D. McGowan, Carlos D. Hunter, Sarah L. Schlichte, Jun Tian, Lynell W. Klassen, James R. O’Dell, Geoffrey M. Thiele, Ted R. Mikuls, Matthew C. Zimmerman, Daniel R. Anderson

**Affiliations:** 1 Department of Internal Medicine, University of Nebraska Medical Center, 982650 Nebraska Medical Center, Omaha, NE 68198-2265, USA; dclemens@unmc.edu (D.L.C.); mduryee@unmc.edu (M.J.D.); andrew.chiou@unmc.edu (A.C.); jake.mcgowan@unmc.edu (J.D.M.); cdhunter@unmc.edu (C.D.H.); lklassen@unmc.edu (L.W.K.); jrodell@unmc.edu (J.R.O.); gthiele@unmc.edu (G.M.T.); tmikuls@unmc.edu (T.R.M.); 2 Veterans Affairs (VA) Nebraska-Western Iowa Health Care System, 4101 Woolworth Ave, Omaha, NE 68105, USA; 3 Fred and Pamela Buffet Cancer Center, Omaha, NE 68114, USA; 4 Department of Cellular and Integrative Physiology, University of Nebraska Medical Center, 982650 Nebraska Medical Center, Omaha, NE 68198-2265, USA; cleofes.sarmiento@unmc.edu (C.S.); sarah.schlichte@unmc.edu (S.L.S.); jtian@unmc.edu (J.T.); mczimmerman@unmc.edu (M.C.Z.)

**Keywords:** doxycycline, oxidative stress, post transnational modification, anti-oxidant, electron paramagnetic resonance

## Abstract

Doxycycline (DOX), a derivative of tetracycline, is a broad-spectrum antibiotic that exhibits a number of therapeutic activities in addition to its antibacterial properties. For example, DOX has been used in the management of a number of diseases characterized by chronic inflammation. One potential mechanism by which DOX inhibits the progression of these diseases is by reducing oxidative stress, thereby inhibiting subsequent lipid peroxidation and inflammatory responses. Herein, we tested the hypothesis that DOX directly scavenges reactive oxygen species (ROS) and inhibits the formation of redox-mediated malondialdehyde-acetaldehyde (MAA) protein adducts. Using a cell-free system, we demonstrated that DOX scavenged reactive oxygen species (ROS) produced during the formation of MAA-adducts and inhibits the formation of MAA-protein adducts. To determine whether DOX scavenges specific ROS, we examined the ability of DOX to directly scavenge superoxide and hydrogen peroxide. Using electron paramagnetic resonance (EPR) spectroscopy, we found that DOX directly scavenged superoxide, but not hydrogen peroxide. Additionally, we found that DOX inhibits MAA-induced activation of Nrf2, a redox-sensitive transcription factor. Together, these findings demonstrate the under-recognized direct antioxidant property of DOX that may help to explain its therapeutic potential in the treatment of conditions characterized by chronic inflammation and increased oxidative stress.

## 1. Introduction

**Figure 1:**
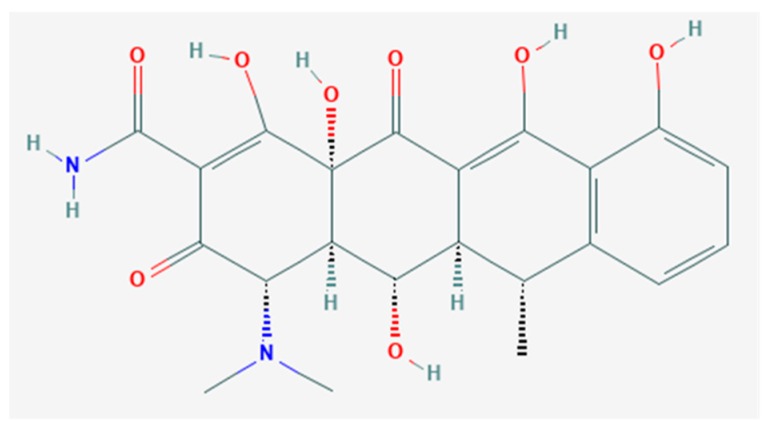
Structure of Doxycycline (DOX) C_22_H_24_N_2_O_8_. Image taken from The National Center for Biotechnology Information. PubChem Compound Database; CID = 54671203, https://pubchem.ncbi.nim.nih.gov/coumpund/54671203 (accessed on 12 December 2018).

Tetracyclines are a family of broad spectrum, well-tolerated antibiotics. Since their discovery over 70 years ago, the modification of naturally occurring tetracyclines and synthesis of novel derivatives has generated numerous compounds. One of the derivatives commonly used clinically is doxycycline (DOX). The chemical structure of DOX is provided in Figure 1. DOX and other tertacyclines exhibit a number of therapeutic properties in addition to their antibacterial activities. These activities include anti-inflammatory, anti-apoptotic, and antioxidant activities [1,2,3,4,5]. Because of these pleotropic actions, DOX and other derivatives of tetracycline have been used in the treatment of a wide variety of diseases. Interestingly, DOX and other derivatives of tetracycline appear to be effective in the treatment of diseases associated with chronic inflammation, including abdominal aortic aneurysms, multiple sclerosis, gingivitis, and rheumatoid arteritis [1,2,3,4,6,7].

Chronic inflammation is commonly associated with oxidative stress. Oxidative stress occurs when the levels of reactive oxygen species (ROS) exceed the capacity of the endogenous antioxidant defense mechanisms. Oxidative stress can initiate inflammatory reactions. Likewise, inflammatory reactions can result in the production of ROS. Because of this, inflammation and oxidative stress, can establish a self-perpetuating cycle resulting in chronic inflammation.

One consequence of the oxidative stress associated with chronic inflammation is lipid peroxidation. Lipid peroxidation in turn, can initiate a self-sustaining reaction resulting in continued lipid peroxidation and perpetuation of inflammation. A major by-product of lipid peroxidation is malondialdehyde (MDA) [8,9]. MDA can spontaneously degrade to form acetaldehyde (AA) [10]. MDA and AA can interact and covalently bind amino groups of proteins and lipoproteins, primarily the ε-amino groups of lysines within these molecules, forming highly stable malondialdehyde-acetaldehyde (MAA) adducts [11,12,13,14]. MAA-adducts are themselves highly immunogenic, initiating robust pro-inflammatory responses providing a link between oxidative stress and inflammation [15,16,17,18]. Therefore, MAA modified proteins and lipoproteins are not only by-products of oxidative stress but may link oxidative stress and inflammation and play a critical role in the pathogenesis of chronic inflammatory diseases.

Considering the well-described link between oxidative stress and chronic inflammation and the fact that DOX has shown benefit in the treatment of some chronic inflammatory diseases, we tested the hypothesis that DOX acts as an antioxidant and directly scavenges ROS to inhibit the production of MAA-adducts. In this report, we demonstrate that DOX is able to directly scavenge superoxide, but not hydrogen peroxide, and inhibits the formation of MAA-adducts. Furthermore, we report that DOX alters intracellular redox signaling as demonstrated by its ability to inhibit the activation of Nrf2, a redox-sensitive transcription factor [19]. The direct antioxidant activity of DOX may be an under-recognized mechanism by which DOX is effective as a treatment for chronic inflammatory diseases. 

## 2. Results

### 2.1. Doxycycline Attenuates MAA-Protein Adduct Formation 

**Figure 2:**
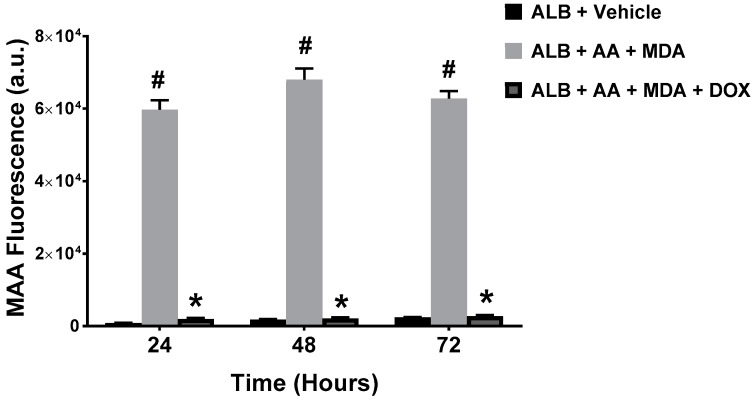
Pharmaceutical Doxycycline (DOX) Inhibits the Formation of MAA-Protein Adducts In Vitro. Human serum albumin (ALB, 1 mg/mL) was incubated at 37 °C with 2 mM malondialdehyde (MDA) and 1 mM acetaldehyde (AA), in the presence or absence of 1 mg/mL pharmaceutical DOX. The formation of MAA-adducted ALB was monitored fluorometrically (excitation 398 nm and emission 460 nm) at 24, 48, and 72 h. The inclusion of DOX significantly inhibited the formation of the MAA-ALB at all time points (* *p* < 0.0001). Increased MAA fluorescence compared to ALB + Vehicle (^#^ *p* < 0.0001) *N* = 10.

To determine whether DOX inhibits the generation of MAA-adducts, we produced MAA-adducts in a cell-free system, by incubating MDA and AA (in a 2:1 molar ratio) with ALB to form MAA-ALB. The formation of MAA-ALB was monitored by its autofluorescence [14]. As shown in Figure 2, the fluorescence of the MDA, AA and ALB reaction increased compared with the ALB and vehicle control reaction. Inclusion of pharmaceutical DOX to the MDA, AA, and ALB reaction significantly attenuated the increase in fluorescence at all time points investigated, indicating decreased formation of MAA-ALB. 

### 2.2. Doxycycline Decreases ROS Produced during the Generation of MAA-Adducts 

**Figure 3:**
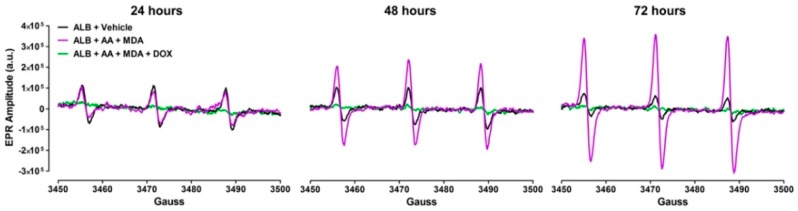
Pharmaceutical Doxycycline (DOX) Decreases ROS Levels Generated by MAA-Adduct Formation. Human serum albumin (ALB, 1 mg/mL) was incubated at 37 °C with 2 mM malondialdehyde (MDA) and 1 mM acetaldehyde (AA), in the presence or absence of 1 mg/mL pharmaceutical DOX for 24, 48, and 72 h. EPR spectroscopy was performed to determine the levels of ROS. Representative EPR spectra are presented. Control samples contained only albumin and vehicle. To detect levels of ROS, all reactions were incubated with 200 µM of the EPR spectroscopy spin probe CMH for the final 30 min of reaction time. a.u. = arbitrary units.

ROS are produced by the interaction of MDA and AA with amino groups of proteins, primarily the ε−amino group of lysines [20,21]. Because DOX attenuated the formation of MAA-ALB (Figure 2), we also investigated whether DOX decreased the levels of ROS produced during the formation of MAA-protein adducts. To accomplish this, ALB was incubated with MDA and AA in the presence or absence of 1 mg/mL pharmaceutical DOX, and ROS were measured by EPR spectroscopy. The results presented in Figure 3 demonstrate that incubation of MDA with AA and ALB produced ROS as demonstrated by the increase in the EPR spectrum amplitude (which is directly proportional to the levels of ROS in the sample) over the 72 h time period. In contrast, inclusion of DOX markedly reduced the increase in the EPR spectrum amplitude, indicating that DOX directly scavenges the ROS produced during MAA-adduct formation. 

### 2.3. Doxycycline Inhibits MAA-ALB-Induced Activation of Nrf2.

**Figure 4:**
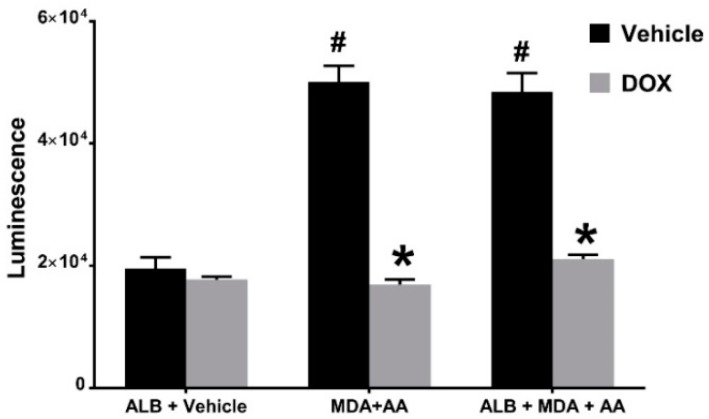
Pharmaceutical Doxycycline (DOX) Inhibits Cellular Redox Signaling Induced by MAA-Adduct Formation. HEK293 cells containing a Nrf2/ARE-responsive element driving luciferase expression were incubated for 24 h in the presence or absence of 1 mg/mL pharmaceutical DOX and either 25 µg/mL ALB, or 2 mM MDA and 1 mM AA, or 25 µg/mL ALB, 2 mM MDA, and 1 mM AA. Significantly decreased with the addition of DOX (* *p* < 0.0001). Significantly increased compared to ALB + Vehicle (^#^ *p* < 0.0001) *N* = 5.

To expand upon the cell-free studies described above and gain insight into whether DOX acts as an antioxidant in biological systems, we utilized HEK 293 Nrf2/ARE cells to investigate the capacity of pharmaceutical DOX to inhibit ROS-mediated activation of Nrf2, a redox-sensitive transcription factor. Culturing HEK 293 Nrf2/ARE cells in media containing MDA and AA, in the presence or absence of ALB for 24 h significantly increased Nrf2 activation, as demonstrated by increased luminescence (Figure 4). Nrf2 activation was significantly attenuated by the inclusion of 1 mg/mL pharmaceutical DOX indicating that the elevated levels of intracellular ROS induced by MDA and AA-mediated formation of MAA-adducts are scavenged by DOX. These results demonstrate that the ROS intermediate of the MAA-adduct or ROS produced during MAA-adduction are able to activate/stabilize Nrf2 and that DOX is able to scavenge these oxidants. 

### 2.4. Pharmaceutical Doxycycline Directly Scavenges Superoxide and Hydrogen Peroxide

**Figure 5:**
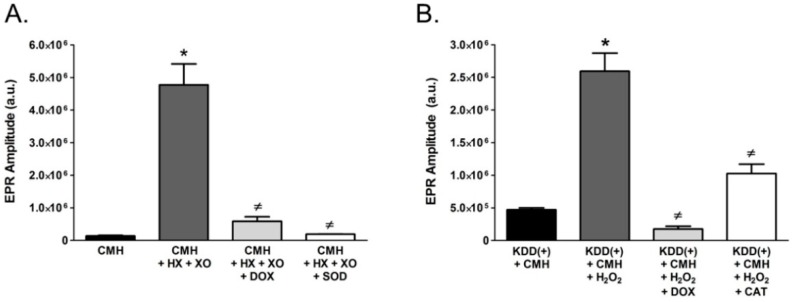
Pharmaceutical Doxycycline (DOX) Directly Scavenges Superoxide (O_2_^•−^) and Hydrogen Peroxide (H_2_O_2_). (**A**) EPR spectroscopy was performed in a O_2_^•−^generating reaction containing 50 μM hypoxanthine (HX), 10 mU/mL of xanthine oxidase (XO), and 200 µM of CMH at 37 °C for 30 min. To determine the ability of DOX to scavenge O_2_^•−^ the reactions were performed in the presence of absence 1 mg/mL pharmaceutical DOX. Control reactions included SOD, a known direct scavenger of O_2_^•−^. (**B**) The ability of pharmaceutical DOX to scavenge H_2_O_2_ was determined by EPR spectroscopy in KDD(+) buffer containing 10 μM H_2_O_2_ in the presence or absence of 1 mg/mL pharmaceutical DOX at 37 °C for 30 min. Control reactions included catalase (CAT), a known direct scavenger of H_2_O_2_. (* *p* < 0.05) vs. CMH (**A**) or KDD(+) and CMH (**B**), (≠ *p* < 0.05) vs. CMH, HX, and XO (**A**) or KDD(+), CMH, and H_2_O_2_ (**B**). *N* = 3–6.

To determine whether pharmaceutical DOX directly scavenges specific ROS, namely O_2_^•−^, we utilized cell-free reactions containing 50 μM HX and 10 mU/mL XO to generate O_2_^•−^, which was measured by EPR spectroscopy. The EPR spectrum amplitude obtained from reactions containing HX, XO, and the CMH spin probe was significantly greater than the amplitude obtained from reactions containing only CMH (Figure 5A). This increase in EPR spectrum amplitude was virtually abolished by pharmaceutical DOX, indicating that pharmaceutical DOX directly scavenges O_2_^•−^. Corroborating the fidelity of the assay, the EPR spectrum amplitude was also abolished by SOD (Figure 5A). 

Next, we investigated whether DOX could directly scavenge other ROS, particularly H_2_O_2_. To accomplish this, we utilized EPR spectroscopy with the CMH spin probe and KDD(+) buffer. As described in the Methods, KDD(+) buffer contains HRP and AAP, which mediate H_2_O_2_-dependent oxidation of CMH to the EPR-detectable stable nitroxide radical [22]. Figure 5B shows that H_2_O_2_ significantly increased the amplitude of the EPR spectrum compared with reactions performed in its absence. Importantly, pharmaceutical DOX dramatically decreased the EPR spectrum amplitude, thus demonstrating that it directly scavenges H_2_O_2_. To ensure the integrity of the assay, separate reactions were performed in the presence of 500 U/mL catalase, an antioxidant enzyme that directly scavenges H_2_O_2_. As expected, catalase also inhibited the H_2_O_2_-induced increase in EPR spectrum amplitude. Unexpectedly, however, pharmaceutical DOX was more effective than catalase at reducing the amplitude of the EPR spectra. 

### 2.5. Ascorbic Acid (ASC) in Pharmaceutical DOX Reduces the CM Radical Spin Probe to Non-Radical CMH 

**Figure 6:**
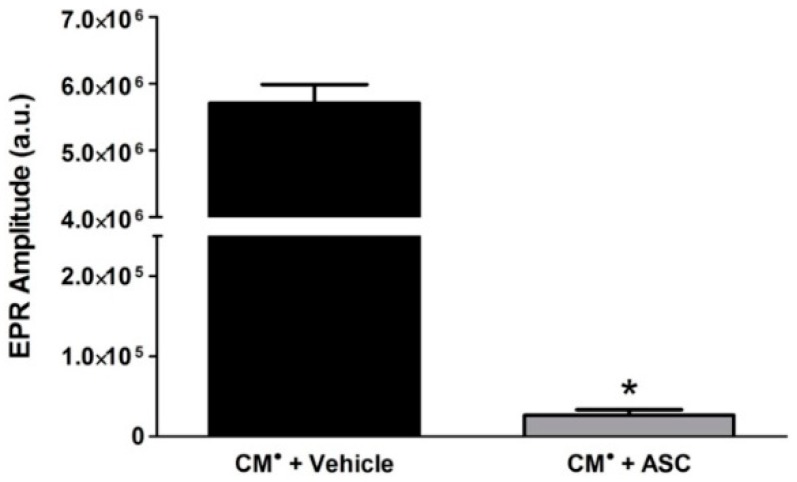
Ascorbic Acid (ASC) Reduces Stable CM^•^ to Non-EPR Detectable CMH Increase. EPR spectroscopy was performed using 200 μM of the stable CM^•^ EPR probe in the presence or absence of 4.8 mg/mL of ASC. (* *p* < 0.0001) vs. CM^•^ and vehicle. *N* = 3.

Because of the unexpected result that pharmaceutical DOX scavenged H_2_O_2_ more effectively than catalase (Figure 5B), we more closely examined the components of the pharmaceutical DOX. Interestingly, we found that the pharmaceutical DOX preparation contains ascorbic acid (ASC) (i.e., Vitamin C), a well-characterized antioxidant [23]. Therefore, to determine the contribution of ASC to the pharmaceutical DOX results (Figure 5), we performed EPR spectroscopy using CM^•^, a stable radical form of the CMH spin probe, and 4.8 mg/mL ASC, which is the concentration of ASC present in the previous reactions containing pharmaceutical DOX. We found that incubation of the stable CM^•^ with ASC abolished the EPR spectrum amplitude (Figure 6), presumably by reducing the stable radical to the non-radical, non-EPR detectable CMH. Thus, the decrease in EPR spectrum amplitude we observed with pharmaceutical DOX is at least partially mediated by the presence of ASC. 

### 2.6. Doxycycline in the Absence of Ascorbic Acid Scavenges Superoxide and Inhibits Intracellular Redox Signaling 

**Figure 7:**
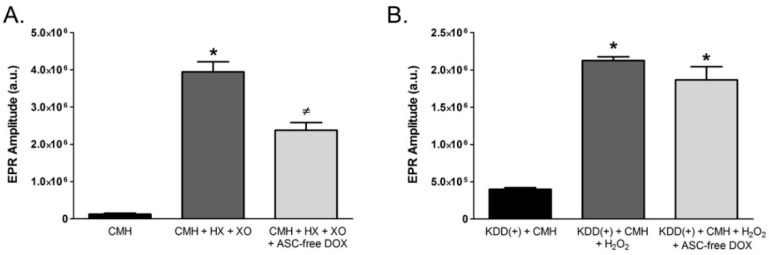
Ascorbic Acid-Free Doxycycline (ASC-free DOX) Directly Scavenges Superoxide. (**A**) EPR spectroscopy was performed in a O_2_^•−^ generating reaction containing 50 μM hypoxanthine (HX) + 10 mU/mL of xanthine oxidase (XO,) and 200 µM of the spin probe, CMH, at 37 °C for 30 min. The ability of ascorbic acid-free DOX (ASC-free DOX) to scavenge O_2_^•−^ was investigated in the presence or absence of 1 mg/mL ASC-free DOX. (**B**) The ability of ASC-free DOX to scavenge H_2_O_2_ was investigated in EPR spectroscopy reactions performed in KDD(+) buffer containing 10 μM H_2_O_2_. Reactions were carried out in the presence or absence of 1 mg/ml ASC-free DOX at 37 °C for 30 min. (*p < 0.05) vs. CMH (**A**) or KDD(+) + CMH (**B**), (≠ *p*< 0.05) vs. CMH+HX+XO (**A**). *N* = 4.

Because ASC reduced the stable EPR spin probe radical to the non-EPR detectable form, we repeated the aforementioned EPR spectroscopy experiments using ASC-free DOX. ASC-free DOX (1 mg/mL) significantly attenuated the EPR spectrum amplitude obtained from cell-free reactions containing CMH, HX, and XO (Figure 7A), indicating that DOX itself is capable of directly scavenging O_2_^•−^. In contrast, ASC-free DOX failed to impact the EPR spectrum amplitude obtained from reactions containing the KDD(+) buffer, CMH, and H_2_O_2_ (Figure 7B), which demonstrates that ASC-free DOX does not directly scavenge H_2_O_2_.

**Figure 8:**
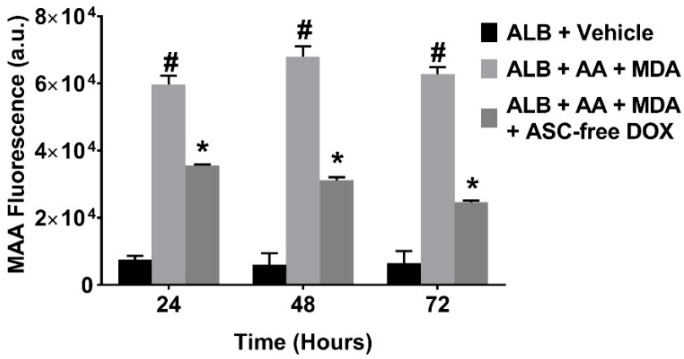
Ascorbic Acid-Free Doxycycline (ASC-free DOX) Inhibits MAA-Protein Adduction. Human serum albumin (ALB, 1 mg/mL) was incubated at 37 °C with 2 mM malondialdehyde (MDA) and 1 mM acetaldehyde (AA), in the presence or absence of ASC-free DOX (1 mg/mL). The formation of MAA-adducted ALB was monitored fluorometrically (excitation 398 nm and emission 460 nm) at 24, 48, and 72 h. The inclusion of ASC-free DOX significantly inhibited the formation of the MAA-ALB (* *p* < 0.0001) vs. ALB + AA + MDA, formation of MAA-ALB was increased (^#^ *p* < 0.0001) vs. ALB + vehicle at the respective time-point. *N* = 4.

To confirm that ASC-free DOX was capable of scavenging ROS, we repeated our MAA-adduct formation assay and investigated whether ASC-free DOX inhibited MAA-ALB generation. ASC-free DOX did indeed inhibit the formation of MAA-ALB at all times points investigated (Figure 8). 

**Figure 9:**
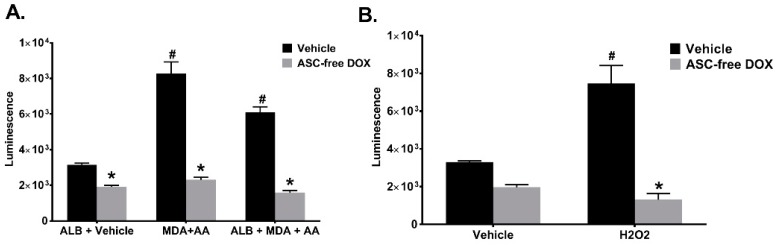
Ascorbic Acid-Free Doxycycline (ASC-free DOX) Inhibits Cellular Redox Signaling Induced by MAA-Adduct Formation. (**A**) HEK 293 cells containing a Nrf2/ARE-responsive element were incubated for 24 h in the presence or absence of 1 mg/mL ASC-free DOX and either ALB + vehicle, MDA + AA, or ALB + MDA + AA. Cells were lysed and luciferase activity determined. Significantly decreased in the presence of DOX (* *p* <0.01). Significantly increased compared to ALB + Vehicle (^#^ *p* < 0.001). (**B**) HEK 293 cells containing a Nrf2/ARE-responsive element were incubated for 24 h with 10 μM H_2_O_2_ in the presence or absence of 1 mg/mL ASC-free DOX. Significantly decreased in the presence of DOX (* *p* < 0.0001). Significantly increased compared to ALB + Vehicle (^#^ *p* < 0.0001). *N* = 4–8.

We also repeated the HEK 293 Nrf2/ARE cell experiments to determine whether ASC-free DOX was able to inhibit Nrf2 activation induced by treatment with ALB, MDA and AA. The results demonstrated that DOX reduced the activation of Nrf2/ARE as indicated by a decrease in luminescence (Figure 9A). Additionally, 1 mg/ml ASC-free DOX was able to attenuate H_2_O_2_-induced Nrf2 activation (Figure 9B). Collectively, these results provide additional evidence that DOX, in the absence of ASC, scavenges specific ROS, particularly O_2_^•−^.

## 3. Discussion

Oxidative stress and inflammation are intimately related [24,25]. Because of this, oxidative stress is a critical factor in the maintenance of inflammation in many chronic inflammatory diseases. Oxidative stress occurs when the levels of ROS, such as O_2_^•−^, ^•^OH, or H_2_O_2_, exceed the capacity of cellular antioxidant systems to reduce them. If not reduced by endogenous antioxidants or scavenging pathways, ROS can cause cellular damage by oxidizing macromolecules such as DNA, proteins, lipids, and lipoproteins. Oxidative stress can also lead to lipid peroxidation, a self-sustaining reaction in which newly formed lipidperoxides are able to react with other lipids resulting in further production of lipidperoxides [13,26,27]. A by-product of lipid peroxidation is MAA-adduct formation. MAA adduction of proteins and lipoproteins has been demonstrated in the diseased tissue of numerous inflammatory diseases, including cardiovascular disease, alcoholic liver disease, smoking-related lung injury, rheumatoid arthritis, and atherosclerosis [15,28,29,30,31,32]. MAA modification of proteins and lipoproteins can affect their function and/or stability and cause cytotoxicity. Importantly, previous studies have clearly demonstrated that MAA-adducts are immunogenic [15,32,33,34]. Thus, MAA-adducts can initiate inflammatory responses, thereby potentially perpetuating the cycle of inflammation and oxidative stress associated with chronic inflammatory diseases [17,35]. 

DOX and other derivatives of tetracycline demonstrate clinical benefit beyond their antibacterial activities [1]. A number of these compounds have been shown to have antioxidant activities [1,36]. Although the exact mechanism by which these compounds act as antioxidants is not known, it has been proposed that the phenol ring of these compounds is central to their ROS scavenging capabilities [36]. It is thought that the reaction of the phenol ring with ROS generates a phenol radical, which is relatively stable and unreactive because of resonance stabilization and steric hindrance by the side groups of the phenolic ring [36]. 

DOX and other tetracyclines such as minocycline have been used in the treatment of gingival disease, abdominal aortic aneurysms, rheumatoid arthritis, acne, neurological disorders such as multiple sclerosis, and have been proposed as a treatment for Alzheimer’s disease [2,3,5,6,37,38]. All of these diseases have a component that is characterized by elevated ROS production and oxidative stress. In preclinical models, DOX has been shown to prevent periodontal disease by inhibiting oxidative stress [39], as well as to provide cardioprotection of hearts during cold storage by balancing oxidant/antioxidant levels [40]. Additionally, in a model of hypertension, DOX treatment reduced aortic matrix metalloproteinase (MMP) activity and ROS levels [41]. 

Because DOX has shown clinical benefit in the treatment of chronic inflammatory diseases and MAA-adducts are associated with many of these diseases, we investigated whether DOX was able to inhibit the formation of MAA-adducts. Using a cell-free system, we demonstrated that DOX inhibits MAA-adduct formation and reduces the production of ROS generated during their formation. Specifically, we demonstrated that DOX directly scavenges O_2_^•−^. We expanded upon these cell-free experiments by also demonstrating that DOX inhibits MAA-adduct-induced intracellular redox signaling, as measured by Nrf2 stabilization/activation. The Nrf2-dependant expression of luciferase was dramatically reduced when DOX was included in the culture media demonstrating that DOX acts as an antioxidant in a biological system. Interestingly, although DOX failed to scavenge H_2_O_2_ in cell-free studies, DOX significantly reduced Nrf2-dependant expression of luciferase induced by H_2_O_2_. These seemingly paradoxical results could stem from the capacity of DOX to scavenge other oxidants, such as the hydroxyl radical or lipid peroxides, produced as a result of the interaction of H_2_O_2_ with other molecules. Thus, DOX is able to moderate free radical levels, and the production of the pro-inflammatory MAA-adducts. It is tempting to speculate that DOX may thereby break the cycle of oxidative stress and inflammation and that this is the mechanism of action by which DOX provides clinical benefit in the treatment of chronic inflammatory diseases.

We have recently shown that methotrexate (MTX), a commonly prescribed medication that has demonstrated benefit in the treatment of chronic inflammatory diseases, is able to act as an antioxidant by scavenging O_2_^•−^ [20]. It has been proposed that the side chains of the phenolic rings of DOX provide it with its antioxidant capability [36]. Inspection of the primary structure of MTX reveals that MTX also possesses side groups that are capable of acting as electron donors. Similar to DOX, we speculate that the antioxidant properties of MTX relate to its side groups, which act as electron donors and directly scavenge ROS. By this mechanism, DOX and MTX could reduce free radicals such as O_2_^•−^ and perhaps other ROS. Furthermore, we suggest that the ability to act as ROS scavengers may be a common mechanism by which water soluble medications such as DOX and MTX provide beneficial, pleotropic activities. 

In summary, we have demonstrated that DOX has direct and specific antioxidant properties, which may explain why DOX and other tetracyclines exhibit clinical benefit beyond their antibacterial properties. DOX is capable of inhibiting MAA-adduct formation, and scavenging ROS generated during their production. More specifically, DOX is able to directly scavenge O_2_^•−^, and can attenuate intracellular redox signaling. Because ROS, oxidative stress, and inflammation are intertwined [42,43], we speculate that, by reducing O_2_^•−^ levels, DOX reduces inflammation, thereby providing benefit in the treatment of chronic inflammatory diseases. Our findings indicate that the non-antibiotic clinical efficacy of DOX observed in the treatment of these chronic inflammatory diseases may be explained, at least in part, by its direct and specific antioxidant properties. 

## 4. Materials and Methods

### 4.1. Malondialdehyde-Acetaldehyde (MAA)-Protein Adduct Formation

MAA-adducted human serum albumin (MAA-ALB) was produced by incubating 1 mg/mL of human serum albumin (ALB) (Talecris Biotherapeutics Inc., Research Triangle Park, NC, USA) with 2 mM MDA, and 1 mM AA, in phosphate-buffered saline (PBS), for 24, 48, or 72 h at 37 °C as previously described [14]. To determine the ability of pharmaceutical DOX (Fresenius Kabi, Lake Aurich, IL, USA) to inhibit the formation of MAA-ALB, 1 mg/mL of DOX was included in these assays.

Additionally, to confirm the role of DOX in these assays, both 4.8 mg/mL ascorbic acid (ASC), which is a component of pharmaceutical DOX, and doxycycline hyclate from Sigma-Aldrich (St. Louis, MO, USA), which is ascorbic acid-free (ASC-free DOX), were tested for the ability to inhibit MAA-adduct formation. The formation of MAA-ALB was monitored by its autofluorescence (excitation 398 nm and emission 460 nm) using a Turner Biosystems (Sunnyvale, CA, USA) LS-5B spectrofluorometer as previously described [14]. 

### 4.2. Electron Paramagnetic Resonance (EPR) Spectroscopy

Using electron paramagnetic resonance (EPR) spectroscopy, we have previously demonstrated that the MAA adduction of ALB produces ROS [20]. Employing this technique, we investigated the ability of DOX to scavenge ROS. Briefly, we measured levels of ROS in 1 mL cell-free reactions containing 1 mg ALB, 2 mM MDA, and 1 mM AA with or without 1 mg/mL pharmaceutical DOX. Control samples contained ALB alone in vehicle (i.e., PBS). Reactions were incubated for 24, 48, or 72 h at 37 °C. After incubation, 200 µM of the EPR spin probe, 1-hydroxy-3-methoxycarbonyl-2,2,5,5-tetramethylpyrrolidine (CMH), was added to each reaction and incubated for 30 min at 37 °C. Fifty microliters of each sample were then loaded into a glass capillary tube and analyzed in a Bruker e-scan EPR spectrometer. ROS produced during the formation of MAA-ALB reacted with the CMH spin probe to produce a stable nitroxide radical (CM^•^), which yielded a characteristic 3-peak EPR spectrum [44]. The level of ROS in the sample was directly proportional to the amplitude of the EPR spectrum [44]. The EPR spectrometer settings used for these and all subsequent experiments were: field sweep width 60.0 G, microwave frequency 9.74 kHz, microwave power 21.90 mW, modulation amplitude 2.37 G, conversion time 10.24 ms, and time constant 40.96 ms.

To determine whether DOX directly scavenges specific ROS, particularly superoxide (O_2_^•−^), cell-free reactions containing hypoxanthine (HX) and xanthine oxidase (XO), which produce O_2_^•−^, were utilized [19]. Samples were prepared with 200 µM CMH spin probe, 50 µM HX, and 10 mU/mL XO with or without 1 mg/mL DOX in 100 µL of EPR buffer (99 mM NaCl, 4.69 mM KCl, 2.5 mM CaCl_2_, 1.2 mM MgSO_4_, 25 mM NaHCO_3_, 1.03 mM KH_2_PO_4_, 5.6 mM d-glucose, 20 mM HEPES) pH 7.4, supplemented with 5 µM and 25 µM of the metal chelators diethyldithiocarbamate (DETC) and deferoxamine, respectively (19). To ensure the fidelity of the assay and confirm the measurement of O_2_^•−^ specifically, control reactions containing CMH, HX, XO, and 400 U copper/zine superoxide dismutase (SOD) were performed. SOD is an antioxidant enzyme that specifically catalyzes the dismutation of O_2_^•−^ into hydrogen peroxide (H_2_O_2_) and oxygen. Samples were incubated at 37 °C for 30 min and the EPR spectra were determined.

Reactions to determine the ability of DOX to directly scavenge H_2_O_2_ were performed with 200 μM CMH and 10 μM H_2_O_2_ in 100 μL of KDD(+) buffer (pH 7.4). KDD(+) buffer is EPR buffer supplemented with 1 mM 4-acetamidophenol (AAP), 1 U/mg horseradish peroxidase (HRP), and 200 μM diethylenetriaminepentaacetic acid (DTPA) [19]. In KDD(+) buffer, HRP and AAP mediate the H_2_O_2_-dependent oxidation of CMH to the stable nitroxide radical [22]. Catalase, a direct scavenger of H_2_O_2_, was used to ensure the fidelity of this assay. Samples were incubated for 30 min at 37 °C and EPR spectra were obtained, as described above. 

### 4.3. Cellular Redox Signaling 

To determine the ability of DOX to modulate cellular redox signaling pathways, which are activated by the generation of MAA-adducts, we employed HEK 293 cells stably transfected with a nuclear factor (erythroid derived 2)-like 2/antioxidant response element (Nrf2/ARE) luciferase reporter construct (HEK293; Signosis, Inc., Santa Clara, CA, USA). In these cells, redox-dependent stabilization of Nrf2 results in its translocation to the nucleus where it binds to the antioxidant response element (ARE) in the promoter located upstream of the firefly luciferase coding region. Thus, stabilization and translocation of Nrf2 to the nucleus leads to its binding and activation of the ARE. Activation of ARE results in luciferase expression, which is determined by measuring luminescence. 

The HEK 293 Nrf2/ARE cells were grown to confluency and incubated with either 1 mg/mL ALB, 2 mM MDA and 1 mM AA, or 1 mg/mL ALB, 2 mM MDA and 1 mM MAA in the presence or absence of 1 mg/mL pharmaceutical DOX or 1 mg/mL ASC-free-DOX for 24 h. After incubation, the growth media was removed and the cells were lysed using a passive lysis buffer as recommended by the manufacturer (Promega, Madison, WI, USA). The luciferase substrate (Signosis) was added to the lysates and luminescence was measured using a Turner Biosystems (Sunnyvale, CA, USA) LS-5B luminometer.

### 4.4. Statistical Analysis

Data are expressed as the mean ± the standard error of the mean (SEM). Statistical analysis was performed using GraphPad Prism 7.04 (GraphPad Software Inc., La Jolla, CA, USA). Significance was determined by one-way ANOVA with Tukey’s post-hoc test where appropriate. Differences were considered significant at *p* ≤ 0.05. 

## 5. Clinical Perspectives

Oxidative stress and chronic inflammation are associated with each other in many different diseases. Previous observations have shown that DOX has some benefits in the treatment of chronic inflammatory diseases. Therefore, we tested the hypothesis that DOX acts as an antioxidant and directly scavenges ROS to inhibit the production of MAA-adducts.

We demonstrate that DOX is able to directly scavenge superoxide, but not hydrogen peroxide, and inhibits the formation of MAA-adducts, which are known products of oxidative stress. 

Reduction of oxidative stress by DOX may reduce inflammation, thereby providing benefit in the treatment of chronic inflammatory diseases. Our findings indicate that the non-antibiotic clinical efficacy of DOX observed in the treatment of these chronic inflammatory diseases may be explained, at least in part, by its direct and specific antioxidant properties. 

## Abbreviations

AA	acetaldehyde
ALB	human serum albumin
ARE	antioxidant response element
ASC	ascorbic acid
CAT	catalase
CM^•^	stable nitroxide radical
CMH	1-hydroxy-3-methoxycarbonyl-2,2,5,5-tetramethylpyrrolidine
DETC	diethyldithiocarbamate
DTPA	diethylenetriaminepentaacetic acid
DOX	doxycycline
EPR	electron paramagnetic resonance
H_2_O_2_	hydrogen peroxide
HX	hypoxanthine
ROS	reactive oxygen species
MDA	malondialdehyde
MAA	malondialdehyde-acetaldehyde
MTX	methotrexate
Nrf2	nuclear factor (erythroid derived 2)-like 2
O_2_^•−^	superoxide
SOD	superoxide dismutase
XO	xanthine oxidase
